# Supplementary material for: Literacy overrides effects of animacy: A picture-naming study with pre-literate German children and adult speakers of German and Arabic
Source: PLoS One. 2024 Apr 17;19(4):e0298659. doi: 10.1371/journal.pone.0298659 (PMC11023297; doi:10.1371/journal.pone.0298659)
Supplement: S1 File — (DOCX) [file pone.0298659.s001.docx]

**S1 Supporting Information**

1. Table 1 provides an overview of noun frequencies. A Welch *t*-test calculated with the SUBTLEX-DE values showed no significant difference between animate and inanimate nouns, *t* = -1.21, df = 57.29, *p* = 0.23. Likewise, a Welch *t*-test revealed no significant differences between animate and inanimate nouns for the childLex values, *t* = -0.16, df = 56.44, *p* = 0.88.

**Table 1. Noun frequencies.**

| **type** | **animacy** | **SUBTLEX-DE**  Mean (SD) | **childLex(6-8)**  Mean (SD) |
| --- | --- | --- | --- |
| **overall** | animate | 2.27 (0.52) | 67.22 (70.25) |
|  | inanimate | 2.42 (0.46) | 64.62 (59.41) |
| masculine,  monosyllabic | animate | 2.43 (0.35) | 60.93 (87.36) |
|  | inanimate | 2.41 (0.59) | 65.28 (82.04) |
| masculine,  bisyllabic | animate | 2.21 (0.52) | 79.21 (82.79) |
|  | inanimate | 2.56 (0.42) | 62.12 (57.26) |
| feminine,  bisyllabic | animate | 2.15 (0.65) | 61.51 (34.3) |
|  | inanimate | 2.28 (0.35) | 66.45 (36.79) |

Frequencies for animate and inanimate nouns in German. The column ‘SUBTLEX-DE’ shows the word frequencies (log10) irrespective of letter case based on subtitles. The column ‘childLex(6-8)’ shows the normalized lemma frequency per million from books for the age groups 6 to 8 years.

1. Summary of the Bayesian hierarchical models. Details on model specification, priors, etc. can be found online at OSF: <https://osf.io/x7n85/>.

In the following tables, we provide the estimate, estimate error and the credible interval for population level effects. The 95% Bayesian credible interval “gives the range over which we can be 95% certain that the true value of the effect lies, given of course the data, the priors, and the model” (Nicenboim & Vasishth, 2016, p. 607). As a rule of thumb, the evidence of an effect is often interpreted as strong if zero lies outside the 95% credible interval (Nicenboim & Vasishth, 2016). Note, however, that this rule of thumb is not always applicable and that credible intervals should not be used to reject the null hypothesis (cf. Nicenboim et al., 2021).

1) Comparison between the German and the Arabic group for filler items in which animacy is matched.

Estimate Est.Error l-95% CI u-95% CI

Intercept (left first responses) 2.33 0.64 1.17 3.71

group1 (German vs. Arabic) -7.22 1.35 -10.18 -4.89

2) Comparison between the German and Arabic group for experimental items in which animacy and position are juxtaposed.

Estimate Est.Error l-95% CI u-95% CI

Intercept (animacy) -0.34 0.36 -1.11 0.32

condition1 (animate left vs. animate right) 6.29 1.52 3.47 9.47

Group1 (German vs. Arabic group) -0.08 0.71 -1.41 1.40

condition1:Group1 -15.80 3.02 - 22.21 -10.35

3) German group: Experimental items in which animacy and position are juxtaposed.

Estimate Est.Error l-95% CI u-95% CI

Intercept (animacy) -1.01 1.25 -3.77 1.26

condition1 (animate left vs. animate right) 15.44 3.07 10.35 22.32

4) Arabic group: Experimental items in which animacy and position are juxtaposed.

Estimate Est.Error l-95% CI u-95% CI

Intercept (animacy) -1.27 0.81 -2.91 0.28

conditionanimate_2nd (animate left vs. right) 1.88 1.72 -1.45 5.32

5) Arabic group: Subsamples of participants who use German at work (GaW) vs. not.

Estimate Est.Error l-95% CI u-95% CI

Intercept (animacy) -0.39 0.37 -1.16 0.34

condition1 (animate left vs. right) -4.45 2.50 -9.38 0.54

GaW (German at place of work vs. not) 0.05 0.47 -0.85 1.00

condition1:GaWGermanatplaceofwork 4.82 3.36 -1.70 11.38

6) German children: Experimental items in which animacy and position are juxtaposed.

Estimate Est.Error l-95% CI u-95% CI

Intercept (animacy) 0.43 0.19 0.07 0.80

condition1 (animate left vs. right) 0.40 0.55 -0.71 1.47

7) Comparison between subsamples of children who have some very rudimentary writing skills vs. those who do not.

Estimate Est.Error l-95% CI u-95% CI

Intercept (animacy) 0.44 0.20 0.04 0.84

condition1 (animate left vs. right) 0.42 0.59 - 0.75 1.60

writing_coded1 (basic writing vs. not) 0.01 0.35 -0.69 0.70

condition1:writing_coded1 0.04 1.17 -2.26 2.34

8) Comparison between German adults and children.

Estimate Est.Error l-95% CI u-95% CI

Intercept (animacy) 0.13 0.27 -0.44 0.65

Group1 (adults vs. children) -0.62 0.52 -1.68 0.35

condition1 (animacy left vs. right) 5.38 0.75 4.01 7.00

Group1:condition1 9.80 1.45 7.19 12.88

1. Results of power analyses. We used the package simr (Green &McLeod, 2016) to conduct a posteriori power analyses for the effects of interest (based on the sample size for the critical comparisons and an alpha-level of .05).
   1. Power for the comparison between the German and the Arabic group for filler items in which animacy is matched (effect of ‘language group’): 100.0% (95% confidence interval: 83.16, 100.0)
   2. Power for the comparison between the German and Arabic group for experimental items in which animacy and position are juxtaposed (interaction effect of ‘position’ and ‘language group’): 100.0% (95% confidence interval: 83.16, 100.0)
   3. Power for the effect of animacy in preschool children (effect of ‘condition’): 15.00% (95% confidence interval: 3.21, 37.89)
   4. Power for the comparison between position and German speaking adults and kids (interaction between ‘position’ and ‘group’): 100.0% (95% confidence interval: 83.16, 100.0)
2. Correlation between children’s age (in months) and their left-first responses


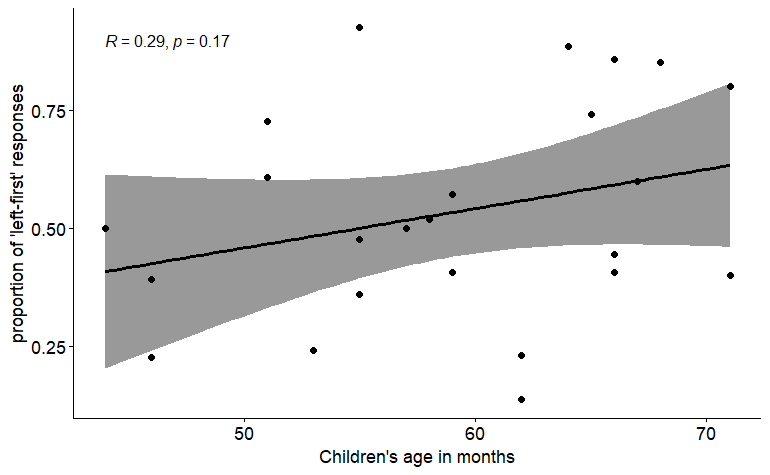


**References**

Green P, MacLeod CJ. “simr: an R package for power analysis of generalised linear mixed models by simulation.” Methods in Ecology and Evolution. 2016;7(4):493–498. doi:10.1111/2041-210X.12504, <https://CRAN.R-project.org/package=simr>.

Nicenboim B, Vasishth S. Statistical methods for linguistic research: Foundational Ideas—Part II. Lang Linguist Compass. 2016;10(11):591–613.

Nicenboim B, Schad D, Vasishth S. An introduction to Bayesian data analysis for cognitive science [Internet]. Under contract with Chapman and Hall/CRC Statistics in the Social and Behavioral Sciences Series.; 2021. Available from: https://vasishth.github.io/bayescogsci/book/
